# Reshaping the Treatment Landscape of a Galactose Metabolism Disorder

**DOI:** 10.1002/jimd.70013

**Published:** 2025-02-14

**Authors:** M. Estela Rubio‐Gozalbo, E. Naomi Vos, Isabel Rivera, Kent Lai, Gerard T. Berry

**Affiliations:** ^1^ Department of Pediatrics, MosaKids Children's Hospital Maastricht University Medical Centre Maastricht the Netherlands; ^2^ European Reference Network for Hereditary Metabolic Disorders (MetabERN) Member Padova Italy; ^3^ United for Metabolic Diseases (UMD) Amsterdam the Netherlands; ^4^ Department of Clinical Genetics Maastricht University Medical Centre Maastricht the Netherlands; ^5^ GROW School for Oncology and Reproduction, Faculty of Health, Medicine and Life Sciences Maastricht University Maastricht the Netherlands; ^6^ Research Institute for Medicines (iMed.ULisboa), Department of Pharmaceutical Sciences and Medicines, Faculty of Pharmacy Universidade de Lisboa Lisbon Portugal; ^7^ Division of Medical Genetics, Department of Pediatrics University of Utah Spencer Fox Eccles School of Medicine Salt Lake City Utah USA; ^8^ Division of Genetics & Genomics Boston Children's Hospital Boston Massachusetts USA; ^9^ Department of Pediatrics Harvard Medical School Boston Massachusetts USA; ^10^ Manton Center for Orphan Disease Research Boston Children's Hospital Boston Massachusetts USA

**Keywords:** aldose reductase inhibitors, classic galactosemia, enzyme replacement, GALK1 inhibitors, nucleic acid therapy, substrate reduction, symptom management

## Abstract

The Leloir pathway was elucidated decades ago, unraveling how galactose is metabolized in the body. Different inborn errors of metabolism in this pathway are known, the most frequent and well‐studied being Classic Galactosemia (CG) (OMIM 230400) due to pathogenic variants in the *GALT* gene. Substrate reduction using dietary restriction of galactose is currently the only available treatment option. Although this burdensome diet resolves the life‐threatening clinical picture in neonates, patients still face long‐term complications, including cognitive and neurological deficits as well as primary ovarian insufficiency. Emerging therapies aim to address these challenges on multiple fronts: (1) restoration of GALT activity with nucleic acid therapies, pharmacological chaperones, or enzyme replacement; (2) influencing the pathological cascade of events to prevent accumulation of metabolites (Galactokinase 1 (GALK1) inhibitors, aldose reductase inhibitors), address myo‐inositol deficiency, or alleviate cellular stress responses; (3) substrate reduction with synthetic biotics or galactose uptake inhibitors to eliminate the need for lifelong diet; and (4) novel approaches to mitigate existing symptoms, such as non‐invasive brain stimulation and reproductive innovations. Early, personalized intervention remains critical for optimizing patient outcomes. We review the advances in the development of different treatment modalities for CG and reflect on the factors that need to be considered and addressed to reshape the landscape of treatment.

## 1 Introduction

**FIGURE 1:**
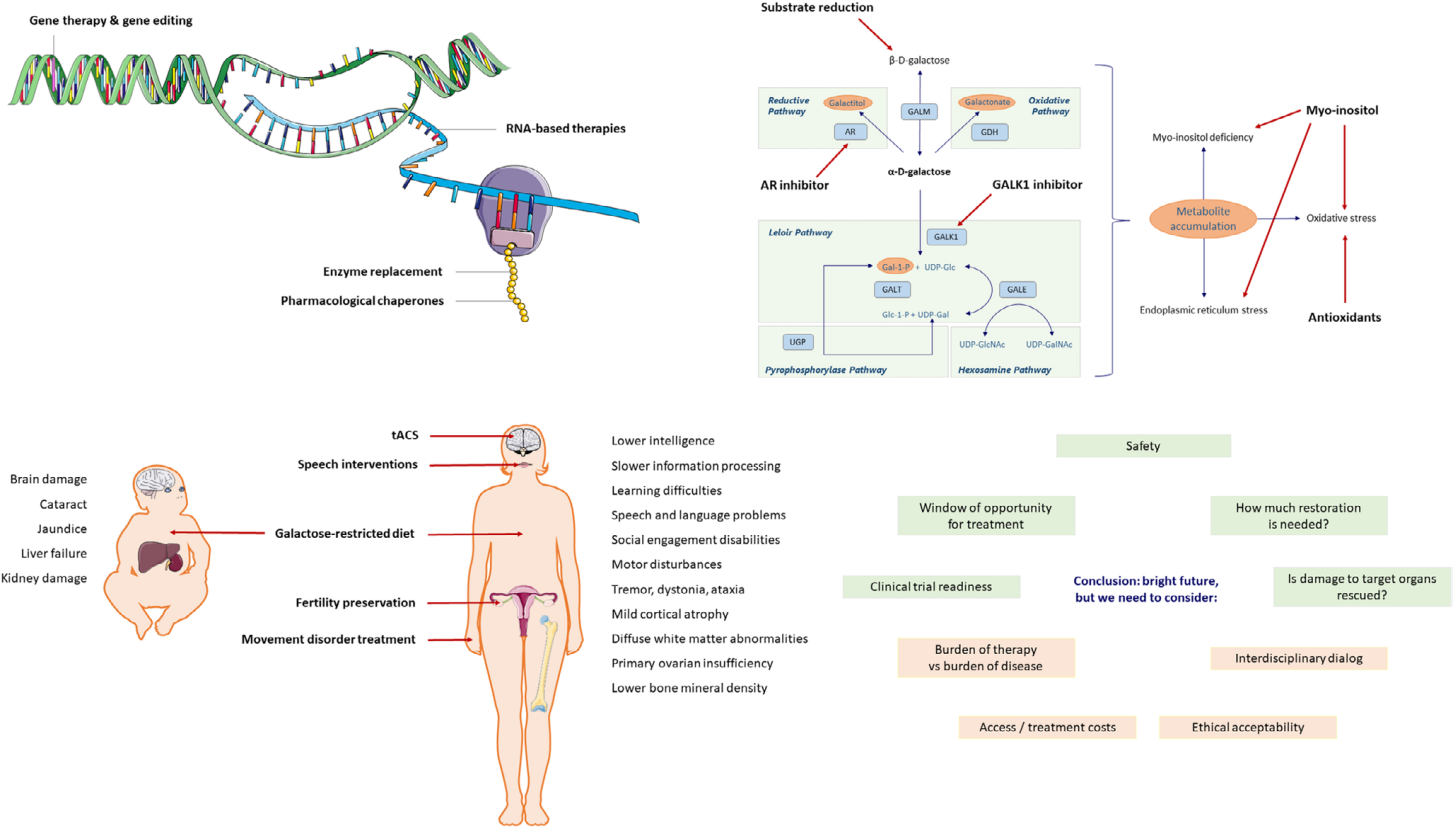
Novel treatment modalities and considerations that reshape the treatment landscape of classic galactosemia. The treatment modalities currently being explored for CG can be grouped in multiple domains: Restoration of enzyme activity (top left), manipulation of the cascade of events (top right), substrate reduction and mitigation of symptoms (bottom left). Before, during and after the design of a new treatment modality, it is important to address several key questions and considerations (bottom right).

In the 1940s and 1950s, a period of biochemistry in which many of the reactions of intermediary metabolism were unraveled, Leloir et al. elucidated the Leloir pathway, which describes how galactose is metabolized in the body. This included the identification of the key enzymes, the discovery of UDP‐sugar intermediates (UDP‐glucose and UDP‐galactose) and the biological significance of the pathway: energy metabolism and biosynthetic purposes (sugar nucleotides for glycoproteins/glycolipids) (Figure 1). Leloir received the Nobel Prize in Chemistry in 1970 for his discovery.

As in other pathways, inborn errors of metabolism due to pathogenic variants in the genes encoding one of the Leloir pathway enzymes are known. The most frequent and well‐studied in this pathway is classic galactosemia (CG) (OMIM 230400) due to pathogenic variants in the *GALT* gene. This gene, located on chromosome 9p13, spans about 4 kb and encodes the protein galactose‐1‐phosphate uridylyltransferase (GALT, EC 2.7.7.12), a 379 amino acid polypeptide homodimer with two active sites (^184^His‐Pro‐His^186^). To date, over 350 (likely) pathogenic *GALT* variants have been reported in ClinVar, with the majority being missense alterations [1]. Residual enzyme activity in CG is absent or barely detectable. Clinical variant galactosemia typically shows enzyme activity between 1% and 10% and a milder natural course. Biochemical variant galactosemia, the most common in the Western world being caused by p.Gln188Arg/4bp deletion‐cis‐N314D (Duarte galactosemia), is a benign condition with residual activity ranging from 15% to 35% [2]. The pathophysiology of CG remains very challenging, and a large number of studies point to various mechanisms being implicated, which may be organ and developmental stage‐specific [3].

Diet, as a substrate reduction strategy, has been the cornerstone for treatment so far. Restriction of galactose intake in the affected newborn infant allows the neonatal life‐threatening multi‐organ disorder to resolve but does not prevent the development of complications such as brain impairments (85%) and primary ovarian insufficiency (80%) [4]. Although it has its benefits, in the neonatal period without any doubt, a lifelong diet is less than ideal and poses a burden for most patients and families who struggle through life having to constantly be aware of not eating dairy milk products. There are also concerns that a too‐strict diet might be harmful [4]. This brings unmet treatment needs to the fore and urges us to apply novel insights and technologies to answer critical questions and develop new treatment strategies. We review the advances in the different treatment modalities and reflect on the factors that need to be considered and addressed to reshape the landscape of treatment.

## 2 Therapy Approaches

Different modalities are under investigation or hypothesized as potential targets and can be grouped as (1) restoring enzyme activity, (2) influencing the cascade of events, (3) substrate reduction other than diet, and (4) novel treatments to mitigate symptoms. While pilot studies yield valuable proof‐of‐concept data, more work is required to establish (1) the optimal window of treatment, (2) dose dependency over a larger range of dosages, (3) the extent of correction for brain‐ and gonad‐related phenotypes, (4) the duration of the therapeutic benefits, (5) disease modifiers that could influence disease outcomes and/or treatment efficacy, and (6) safety.

### 2.1 Restoring GALT Activity

Therapeutic approaches that aim to restore the missing enzyme activities in recessive, monogenic diseases are gaining popularity because they theoretically address the root causes of the disorders and, therefore, offer the patients a modality that comes very close to a cure. In accordance with the international clinical guideline for the management of classical galactosemia, patients with more than 15% residual enzyme activity do not require treatment [5]. As such, restoration of 10%–15% enzyme activity would make a desirable target for these treatment modalities.

#### 2.1.1 Gene Therapy

The first documented introduction of the human *GALT* gene replenishing GALT expression in GALT‐deficient patient cells was carried out by Slepak et al. [6]. In that study, they used a lentiviral vector expressing the human *GALT* gene to infect primary skin fibroblast strains from patients harboring homozygous c.563 A>G; p.Gln188Arg mutations in the *GALT* gene. As isogenic controls, they used lentiviral vectors expressing the green fluorescent protein (GFP). The authors not only showed restoration of galactose metabolism in the GALT‐reconstituted cells but also the manifestation of an unfolded protein response (UPR) in the control GALT‐deficient cells. Despite the interesting results, the authors did not advocate further development of the lentiviral vector as a therapy due to the fear of potential detrimental consequences of genomic integration of lentivectors [7, 8].

Following the early documented discoveries of adeno‐associated virus (AAV) [9, 10, 11], research has quickly capitalized on the use of recombinant AAV‐based viral vectors in the delivery of transgenes (gene replacement) [12] or the molecular components of the gene editing system [13, 14] as emerging vectors for nucleic acid (DNA‐ or RNA‐based) therapies for many diseases.

Although the limited size of the cargo DNA is a known weakness of the AAV‐based vectors [15], this does not pose any challenge for CG because *GALT* cDNA is relatively short and can be easily packaged into the recombinant AAV vector. Moreover, compared with lentivirus, the cancer‐causing risk for recombinant AAV integration is minimal. First, most recombinant AAV vectors exist as episomes [15]. Second, even when they integrate, two recent large companion studies in nonhuman primates indicate that vector integrations in animal liver following AAV gene therapy may be an important mechanism for achieving durable expression and are unlikely to induce cancer mutations in humans [16, 17]. Most AAV serotypes have wide tissue tropism, which is advantageous because multiple organ systems are affected in Classic Galactosemia. For instance, AAV9‐ and AAVrh10‐based vectors can pass through blood‐brain barriers, making them preferred vectors to tackle neurological complications.

At least three groups have documented the use of AAV‐based vectors derived from different serotypes (AAV2, AAV8, AAV9, and AAVrh10) in their pilot studies in patient cells and GalT‐deficient rodent animals [18, 19, 20]. Not surprisingly, the expression of the human *GALT* transgene, which is under ubiquitous promoters, resulted in abundant GALT protein synthesis and significant reduction of abnormal galactose metabolites in selected organs (brain, liver) and blood, and in one report, restored whole body galactose oxidation [18].

Like all systemic AAV‐based therapies (approved or experimental), systemic AAV‐based gene replacement treatment for CG, if approved, is likely to face the same challenges of immunogenicity and genotoxicity, and related side effects [21, 22, 23]. The immunogenic responses (innate and adaptive) arise when the host immune system encounters both the viral coat (capsid) and the recombinant vector and the transgene. The adaptive response also leads to the production of neutralizing Ab, which prevents repeated dosing in the future. Novel strategies are being developed to mitigate immunogenicity. Strategies applicable to Galactosemia might include (a) dosage reduction and (b) improved vector design. The former, which can help reduce immune response and minimize off‐target effects, can be achieved through a hyperactive variant of the transgene as well as tissue‐specific promoters that aim to reduce exposure to the transgene product for the host immune system. It can also theoretically be achieved if we can exploit the unique tissue tropism of different AAV serotypes and target certain cell types that are deemed to render significant clinical benefits for the complications suffered by the patients. For improved vector design, one will require the identification and/or design of capsid variants that are less immunogenic as well as the minimization of the empty capsid, which is a critical quality control issue for GMP‐grade vector manufacturing [24, 25, 26].

Unlike gene replacement, which enjoys vast popularity among translational scientists as a preferred therapeutic approach for monogenic disorders, gene editing using CRISPR/Cas9 or similar approaches has just begun to establish a foothold in the therapeutic arena. The precise and targeted modification of genetic material is a most significant advancement. Off‐target editing remains a concern, and efforts to predict and detect off‐target edits, improve editing specificity, and generate novel delivery systems are being made. For CG, as the c.563A>G; p.Gln188Arg variant allele represents up to 70% of all galactosemic alleles, one may propose that if successful and safe, gene editing techniques that target this specific variant could bring substantial benefits to a significant number, albeit not all, of the patients [27]. This novel approach has yet to be studied for CG.

Instead of viral vectors, one can in theory deliver *GALT* cDNA using nanoparticles; so far, lipid nanoparticles (LNP) are mostly being used [28]. However, the LNP design needs to exhibit the right tissue tropism. For instance, follicle stimulating hormone (FSH) peptide‐conjugated nanoparticles [29] have been used to deliver shRNA to target ovarian cancer cells. Whether this approach can be used in CG remains to be seen. Apart from synthetic LNPs, a class of particles called virus‐like particles (VLP) has also been employed as a delivery vector for nucleic acid modalities [30, 31, 32]. VLPs mimic the structure of viruses and retain their cell‐targeting capacity but are devoid of the viral DNA or RNA and thereby lack the viral risk of oncogenesis. It is therefore possible to exploit this approach to deliver plasmid expressing *GALT* cDNA, genome editors, or *GALT* mRNA. VLPs can elicit immune responses from the host, which makes it more challenging for repeated dosing but could be employed to deliver *GALT* gene cDNA or genome editors that have a longer expression half‐life. Like all nanoparticles, their molecular size and tissue tropism will ultimately dictate how successful they can be in their applications in disease treatments.

#### 2.1.2 RNA Therapy

In addition to DNA‐based molecular therapies, RNA‐based experimental therapies using lipid nanoparticles (LNP)‐encapsulated in vitro transcribed (IVT)‐mRNA, circular RNA (cRNA) [33], and self‐amplifying (saRNA) [34, 35] are being explored, especially after the clinical success of mRNA‐based COVID‐19 vaccines [36, 37]. Compared to the accelerated regulatory approval of the COVID‐19 vaccines, no RNA‐based therapies for inborn errors of metabolism (IEM) have been approved, despite a few clinical trials using IVTmRNA being conducted for IEMs like methylmalonic acidemia (NCT04899310) and glycogen storage disease type 1a (NCT05095727). Nevertheless, proof‐of‐concept studies using LNP‐encapsulated IVT‐*GALT* mRNA have been carried out in model organisms [18, 38, 39]. Restoration of galactose metabolism, re‐expression of GALT activity, and reduction of toxic galactose metabolites were seen in all reports. The half‐life of the IVT‐*GALT* mRNA has seen significant improvement throughout the years from 3 days to 14 days [18, 38], but it still falls short of the significantly longer half‐lives of *GALT* expression, which usually last longer than 6 months, seen in the AAV‐based gene replacement studies. The tropism of LNP used in the studies may pose limitations for the range of target organs that would need *GALT* augmentation. Future work should focus on the correction of disease‐relevant phenotypes and long‐term safety evaluation.

#### 2.1.3 Enzyme Replacement

Enzyme replacement therapy (ERT) has successfully been employed for various inborn errors of metabolism, especially storage disorders, but can in theory be customized to benefit any disease caused by an enzyme deficiency, including galactosemia. As current ERT modalities are unable to cross the blood–brain barrier (BBB), novel nanoparticles are being engineered to address this problem, such as fusion proteins that can bind to receptors on the BBB to facilitate their uptake [40]. Specifically for CG, VLPs derived from the plant brome mosaic virus (BMV) have been developed as a new form of enzyme replacement and were able to deliver GALT protein in fibroblasts, hepatocytes, and kidney cell lines [41]. Future studies should tell us if this method is successful in decreasing the levels of galactose and its metabolites.

#### 2.1.4 Chaperones

Most *GALT* pathogenic variants, including the highly prevalent c.563A>G; p.Gln188Arg, are missense variants that give rise to proteins that are prone to misfolding—a critical process for achieving their functional three‐dimensional conformation. Protein folding is facilitated by molecular chaperones, small proteins that assist in proper folding. This has led to the hypothesis of using chemical or pharmacologic chaperones to correct defective folding processes. Arginine, known for its anti‐aggregation properties, has shown promise in some pathologies [42, 43]; however, results regarding its effect on the c.563A>G (p.Gln188Arg) GALT variant in vitro and in vivo studies are conflicting [44, 45]. Beyond misfolding, this specific variant's location in the active site likely disrupts substrate interactions [46]. Investigating chaperone strategies for this and other GALT variants remains an open and promising area of research. Notably, new findings have recently been published [47].

### 2.2 Influencing the Cascade of Events

A severely diminished GALT enzyme activity gives rise to a cascade of events and renders several intervention possibilities. These approaches can each tackle one of the different aspects involved in pathophysiology and may be used as a combination in the future.

#### 2.2.1 GALK1 Inhibitors

GALT deficiency originates the accumulation of Gal‐1‐P and is known to play a fundamental role in classic galactosemia pathogenesis; on the other hand, patients suffering from GALK1 deficiency (OMIM 230200), who accumulate several galactose metabolites but not Gal‐1‐P, present mild symptoms, essentially early‐onset cataracts but not brain and ovarian complications, the major and until now irresolvable problem affecting GALT‐deficient patients. Accordingly, GALK1 enzyme (EC 2.7.1.6) inhibition to reduce substrate accumulation was envisaged as a good therapeutic target. Nevertheless, GALK1 is a small molecule belonging to the GHMP kinase superfamily of ATP‐dependent enzymes involved in the biosynthesis of isoprenes and amino acids as well as in carbohydrate metabolism. The first GALK1 inhibitors were ATP‐competitive, thus affecting also the function of other GHMP kinases; recently, Yue and colleagues are developing non‐orthosteric compounds that do not target the ATP and galactose binding sites, thus increasing the specificity and potency of GALK1 inhibition [48].

#### 2.2.2 Aldose Reductase Inhibitors

Another toxic metabolite in GALT deficiency is galactitol, which is formed by the metabolism of galactose by aldose reductase (AR, EC 1.1.1.21), an NADPH‐dependent enzyme. Galactitol accumulation inside cells causes their swelling and apoptosis, causing cataracts; moreover, high levels of galactitol were also detected in the brains of GALT‐deficient patients [49], which may play a role in the development of neurological symptoms, causing injury in Schwann cells [50]. So, the development of AR inhibitors constitutes another therapeutic target, aiming to decrease galactitol levels in tissues.

Phase 1/2 clinical trials involving adult patients with GALT deficiency have already been performed to test the effect of AT‐007 (Govorestat), an oral molecule able to cross the BBB, and the results revealed its safety and effectiveness in significantly lowering galactitol plasma levels [51]. A very recent study involving pediatric patients (2–17 years old) revealed that Govorestat treatment, besides lowering galactitol levels as previously proved, reported a positive effect on some clinical measures [52]. So far, AT‐007 has not received FDA approval.

#### 2.2.3 Myo‐Inositol and Its Supplementation

Myo‐inositol (mI) was first reported to be reduced in the brain of patients with galactosemia post‐autopsy [53]. This was initially confirmed in an MRI/MRS study by Berry et al. [49] and thereafter in several additional reports [54]. mI has several biological properties, such as regulating Ca2+ signaling and acting as an osmolyte. It is converted into phosphatidylinositol at the ER membrane and transported to the plasma membrane, where it is phosphorylated at one or multiple sites to form one of the phosphatidylinositol‐derived species. The phosphoinositides serve a plethora of important biological functions, including (endosomal) vesicle trafficking, autophagy, cytoskeletal reorganization, lipid homeostasis, regulation of ion channel and transporter activity, and activation of calcium and protein kinase C signaling [55]. There is a dual mechanism to explain its deficiency in CG: galactitol inhibits the SMIT1 transporter at the plasma membrane responsible for the transport of extracellular myo‐inositol into the cytoplasm. In addition, Gal‐1‐P interferes with myo‐inositol monophosphatase (IMPase; EC 3.1.3.25), responsible for endogenous myo‐inositol synthesis. The higher the levels of galactitol and Gal‐1‐P, the more prominent the role in the pathophysiology of this mechanism. Supplementation of mI to the mouse model showed a positive effect on gonadal and brain damage [56].

#### 2.2.4 Antioxidants/Endoplasmic Reticulum Stress/Integrated Stress Modulators

There is evidence that oxidative stress and ER stress are mechanisms involved in CG damage [56, 57]. The ovaries and brain are particularly vulnerable to both oxidative stress and ER stress due to their high metabolic activity and limited capacity to manage cellular stress. In the brain, high oxygen demand, lipid‐rich membranes, and glutamate excitotoxicity exacerbate oxidative and ER stress. In fact, there is ample evidence for the implication of excessive oxidative stress in different animal/cell models of CG [3, 56, 57, 58, 59, 60, 61, 62], and it has been suggested that antioxidants could be employed to ameliorate the symptoms of this disorder [56, 63].

Purple sweet potato color (PSPC) supplementation has shown promise in addressing complications of classic galactosemia in a GALT‐deficient mouse model [56]. PSPC, known for its strong antioxidant and anti‐inflammatory properties, was found to improve cellular health in organs like the brain, ovary, and liver. It reduced oxidative stress and addressed dysregulated pathways associated with galactosemia, including stress responses in ovarian and neuronal tissues. Additionally, PSPC supplementation showed the potential to mitigate markers of cellular stress, such as the integrated stress response and ER stress, which are often elevated in galactosemic conditions.

These modalities, mI and cellular stress reducers, are appealing. They have a favorable safety profile, are well‐tolerated, cross the BBB, can be taken in the outpatient setting, are non‐invasive, and offer a cost‐effective alternative due to relatively simple extraction and formulation processes compared to sophisticated biotechnological methods required for example gene therapies. Human studies are needed to confirm their efficacy and optimal dosing. Since damage in this entity is very likely to at least partly have a prenatal origin, being able to intervene during gestation with these supplements might offer the best possible outcomes. The administration of mI during pregnancy has been well‐documented and deemed safe, while PSPC requires further research. Exploring the use of these therapies in clinical trials seems a logical next step.

### 2.3 Substrate Reduction Other Than Diet

Intestine and kidney galactose transport inhibitors and gut‐restricted synthetic biotics are theoretically possible, but no studies have been performed yet. These approaches would possibly replace the lifelong diet and might be important for the quality of life of patients who struggle with their diet. However, this approach is not likely to influence the natural course of the disease.

Galactose restriction, other than diet, implicates its impaired absorption to the kidney and the small intestine by the Na^+^‐glucose co‐transporter 1 (SGLT1) and to the systemic side by glucose transporter 2 (GLUT2). Inhibition of these transporters has already been studied mainly for type 2 diabetes mellitus (OMIM 125853) treatment [64].

Synthetic biotics are microorganisms, typically bacteria, that have been genetically modified to serve specific therapeutic or industrial purposes. They could, for example, be engineered to break down harmful substances in the gut to prevent their absorption into circulation. Synthetic biotics have been developed for several pathologies, including metabolic diseases like phenylketonuria (OMIM 261600) [65] and may have a place in future galactosemia treatment strategies.

### 2.4 Advancing Symptomatic Treatments

Language and speech problems are very common in CG. Studies from Nancy Potter's group explore clinical management focused on cognitive and early speech and language skills during ages as early as < 6 to 24 months to achieve better outcomes [66].

Even when symptoms have occurred, strategies to mitigate them might still be very beneficial to the patients. Also here, there are new technologies and developments to exploit. Recently, altered neural oscillations in a small group of classic galactosemia patients during sentence production were described [67] offering a rationale for transcranial alternating current stimulation aiming to restore the altered neural oscillations and improve language performance [68]. The results showed that theta stimulation, not sham, significantly reduced naming error percentage in patients, not in controls. Theta did not systematically speed up naming beyond a general learning effect, which was larger for the patients. The studies so far are limited but promising and could be extended using different stimulation frequencies and sites of stimulation to explore their potential in enhancing visuospatial memory or combating anxiety in these patients.

Movement disorders have a significant impact on the lives of a subgroup of patients. Recommendations to better characterize the movement disorder in the affected individual and therapy advice based on the different components of the movement disorder would also be helpful and warrant attention.

Ovarian tissue cryopreservation (OTC) is a key technique, where ovarian tissue is harvested and preserved, with the possibility of future use if fertility becomes a concern later in life. This procedure has been applied successfully to young girls with CG, offering hope for future reproductive options despite early ovarian failure [69, 70]. As spontaneous pregnancy occurs in CG, despite primary (or premature) ovarian insufficiency (POI), one may tentatively assume that the cryopreserved tissue will lead to a pregnancy and child, but there is no evidence yet that this will be the case.

The examples mentioned above are just a few ways in which novel advancements and insights can positively impact the burden of disease, highlighting an area that requires attention.

## 3 Window of Opportunity for Treatment(s)

To develop an effective treatment strategy, it is important to understand at which time‐point damage occurs so that the treatment can be initiated on time to optimize long‐term outcomes. It might be that for treatments to be curative, prenatal intervention is needed. However, data gathered so far suggest that **early** postnatal intervention is also able to positively influence the natural course of this disease [38, 56, 71]. This seems to make sense as, although damage might start prenatally, the ovary and brain are two very dynamic organs in which processes important in the prenatal period continue after birth and are still amenable to interventions (neurogenesis, myelination, network forming to name several in the brain, as well as programmed cell death in the ovary).

When a treatment modality is meant to prevent the manifestation of long‐term complications, such as those that would restore GALT activity, it is important to instigate treatment as soon as possible, if not prenatally. For the brain, damage likely originates early in life [72], meaning that treatment may have to start prenatally or early in infancy to optimize speech and cognitive outcomes later in life. Some symptoms, such as tremor, are more common at an older age; some argue that this is the result of continuing damage or an early hit with a dying back phenomenon [72]. Also, neurological systems compensate or adapt during development, which might delay symptom onset. Tremors could appear as the brain's capacity to compensate declines or as demands on the motor system increase with age. For the ovaries, treatment to halt the rapid depletion of follicles is most likely necessary in early childhood to protect the ovarian reserve that is still relatively preserved at this age [70].

For therapies that are not aimed at damage prevention, but rather at mitigating pre‐existing symptoms, the window of opportunity will be much wider. When determining the optimal window of opportunity for a specific treatment modality, it is also important to consider the patient's characteristics. Disease modifiers could be used in predictive models for long‐term complications, which may improve patient care and disease outcomes with a more personalized treatment. Nucleic acid therapy can be arguable for a patient who will be very mildly affected, but might prove life‐altering for patients who are predicted to battle with severe long‐term complications.

The identification of (genetic) modifiers that may contribute to the outcome, can help us delineate the “window of opportunity” for different treatment modalities. However, it is good to keep in mind that this window may depend on the nature of the treatment modality as well as the patient's characteristics.

Differences in brain and ovary susceptibility due to modifier genes, polygenic and epistatic interactions regulating responses to oxidative stress, ER stress, apoptosis, and inflammation could be responsible for the observed clinical heterogeneity. In a child with an unfavorable genetic background, the window of opportunity would shift to as early as possible. These predictive disease modifiers do not necessarily have to be (epi)genetic in nature. For example, girls are born with a follicle reserve ranging from 500.000 to 2 million primordial follicles [73] due to multiple factors including the maternal environment. As a logical consequence, a female CG patient born with 2 million follicles will have more time before her ovarian reserve runs out and therefore has a better chance at achieving spontaneous menarche or even non‐assisted pregnancy than a patient who only had 500.000 follicles to start with.

## 4 Other Considerations

Researchers should prioritize treatment modalities not only based on their efficacy to reduce long‐term symptoms but also based on the ethical and social‐economical considerations that come with it. Myo‐inositol or PSPC are two examples of low‐cost treatments that could be made accessible even to patients in countries with underdeveloped health care systems, and given their safety profile, would be much easier to implement as standard of care than, for example, nucleic acid therapies. Therapies with less favorable risk–benefit ratios or higher treatment costs may only be approved for patients who are predicted to face severe long‐term complications if not treated. Interdisciplinary dialogue among healthcare professionals, ethicists, and patient advocacy groups is paramount to ensure that novel treatment modalities are not only effective but also cost‐effective and ethically sound.

## 5 Conclusion

Patients and families are desperate for better treatment options, and they feel the progress being made in this complex disease is very slow. Yet, while many years ago the only available intervention was diet, a new landscape has emerged with the advancement in technologies and insights, and the future seems brighter. For a given therapy, we will need to consider the burden and risks of therapy versus the burden of disease, the access to treatment, the costs, the ethical issues, the clinical readiness, as well as the patient voices on new developments. With many modalities ready to enter clinical trials, an important topic is clinical readiness, that is, having clinical outcome measures and biomarkers that truly mirror the effectiveness of the intervention. Efforts need to be made to achieve this goal, which is one of the Galactosemia Network members' priorities for the coming period (www.galactosemianetwork.org).

## Author Contributions

**M.E.R.‐G.:** conceptualization. **M.E.R.‐G, E.N.V., I.R., K.L.,** and **G.T.B.:** writing – original draft preparation. All authors have read and agreed to the published version of the manuscript.

## Conflicts of Interest

The authors declare no conflicts of interest.
